# Fever and Diarrhœa from Foul Water

**Published:** 1859-01

**Authors:** W. I. Cox


### FEVER AND DIARRHŒA FROM FOUL WATER.

By W. I. COX, M.R.C.S., etc.

In the October number of this Review, I spoke at large of the obvious and proven connection of epidemic maladies with the use of foul water and the neglect of proper sewage. Since the writing of the remarks alluded to, cases have occurred in the vicinity of my residence, offering additional and striking illustration of the facts I then brought forward. These I will briefly relate.

I must premise by stating that, with the exception of those about to be mentioned, no cases of diarrhœa or fever have occurred in my district during the last half year. The village wherein I live has remained quite free from those maladies up to the present time. About a mile from the village is a piece of waste-land (common) on which stand ten or twelve houses; and these, with one exception, have no water-supply, save that obtained from surface-pools. Owing to the unusual dryness of the past season, even this source had become less available and more loathsome than wont. Several cases of very severe diarrhœa occurred, among the people inhabiting these houses, in the beginning of September; the children especially were violently affected. On the 20th of that month, two adults, inhabiting the house mentioned above as forming an exception in favour of water-supply, were suddenly attacked with symptoms so nearly approaching to those characterising Asiatic cholera, that, had that epidemic prevailed at the time, I should, unhesitatingly, have set them down as cholera cases. In one case, especially that of Mrs. C., the mistress of the house, a strong healthy woman of 30 years of age, there were rice-water purging, excessive vomiting, sunken features, absolute failure of the pulse at the wrists for ten hours, coldness and lividity of extremities, and croaking voice. The other patient, the husband's mother, was also in a state of severe collapse for some time. Both ultimately recovered with the adoption of energetic treatment. Two children were also attacked in the same house a few days afterwards, but their symptoms were not of so alarming a character. On inquiry, I found that the well on the premises from which they obtained the water for domestic purposes had, about a month previous to this outbreak, become unavailable through some accident, and they had been compelled to use for the house water obtained from a filthy pool at some distance. I examined some of this water. It was so charged with organic decomposing matters (evidently from contamination with soil-drainings,) that the presence of hydrosulphuret of ammonia was readily shown in it by the usual simple tests. It seems necessary to add that the family were by no means poor, but of cleanly, well-ordered habits, and healthy constitutions.

In a village, situate about two miles from that wherein I live, has lately occurred a case of *continued fever*, terminating fatally from ulceration of the intestinal glands, and which seems to me to have arisen indubitably from sewer emanation, and from no other source whatever. The victim was a young man about 30 years of age, previously in good health, although of a weak constitution. He had employed himself in cleaning out a cesspool at the back of his premises about five weeks before the onset of his malady. At the time of his doing so he felt sick, and, as he expressed it, "dull and head-achy," which sensations never wore entirely off until the characteristic rigors and sleeplessness commenced. No other case of fever has occurred in the village or neighbourhood for the past nine months. On the other hand, the poor man in question had not visited any spot at a distance where fever was prevalent. Indeed, his family assured me that he had not been out of the village for some months previously. Now in this case, was not the fever poison generated from the cesspool emanations? It seems to me quite impossible to come to any other conclusion, or to regard the matter in any other light than as direct cause and effect. Neither, in fact, of the instances I have given can be esteemed as mere fortuitous coincidences.

I am sure I need not apologise for again, and so soon, trespassing on the columns of this REVIEW with regard to such matters. It seems to me important for medical observers to record every mite of positive fact and true logical deduction bearing on these important subjects.

Hawkesbury Upton, Gloucestershire,
November 1858.

